# Siefken, Sven T. (Hrsg.) (2021): *Wahlkreisarbeit von Bundestagsabgeordneten. Parlamentarische Repräsentation in der Corona-Krise*: Wiesbaden: Springer VS. 150 Seiten. 54,99 €

**DOI:** 10.1007/s11615-022-00449-9

**Published:** 2023-01-11

**Authors:** Nadin Fromm

**Affiliations:** Fachgebiet Public Management, Universität Kassel, Kassel, Deutschland

Wir haben das Jahr 2019. Unerwartet erkranken Menschen an einer bisher unbekannten virusbedingten Lungenkranheit in Wuhan, in der zentralchinesischen Provinz Hubei. Kurze Zeit später werden auch außerhalb Chinas Krankheitsfälle gemeldet. Die Weltgesundheitsorganisation deklariert die Ausbreitung der COVID-19-Infektionskrankheit zur weltweiten Pandemie. Es zeigt sich, dass diese Krisensituation die Vorzeichen staatlichen Handelns transformiert. Die Folge ist eine Ausweitung und neue Qualität der Staatstätigkeit. Das Verhältnis von Politik und Bevölkerung, insbesondere das von Repräsentant:innen und Repräsentierten, ist davon betroffen und unterliegt gravierenden Veränderungen.

Diese u. a. aus verwaltungs- und politikwissenschaftlicher Sicht als einschlägig zu bewertenden Transformationsprozesse geben den Ausschlag für die Entstehung des vorliegenden Buches. Der Herausgeber *Sven T. Siefken*, ehemals Dozent an der Martin-Luther-Universität Halle-Wittenberg, plant ursprünglich im Rahmen eines Forschungsseminars die Auswertung von Daten aus Interviews mit Abgeordneten der nationalen Parlamente Deutschlands und Frankreichs. Die Interviewdaten sind Datenbasis des abgeschlossenen Forschungsprojektes „Citizens and Representatives“, das in Kooperation mit Politikwissenschaftler:innen der Universitäten Halle, Stuttgart und dem Institut d’études politiques, Bordeaux (Frankreich), durchgeführt wurde. Im Fokus steht die Wahlkreisarbeit von Abgeordneten, vor dem Hintergrund ihrer Wahrnehmung der Aufgaben als Interessensvertretung der Bürger:innen. *Siefken *eröffnet den Teilnehmer:innen des genannten Forschungsseminars die Gelegenheit, die Auswertungsmethoden qualitativer Sozialforschung anhand dieser Forschungsdaten zu erlernen und zu erproben. Er entscheidet sich jedoch angesichts der COVID-19-Pandemie spontan dazu, das Seminar thematisch auf die Erhebungsmethoden qualitativer Sozialforschung auszuweiten. Die eingangs skizzierte Problemstellung dient als Ausgangspunkt für die Interviewstudie. Schwerpunktmäßig geht es nun um das Rollen- und Repräsentationsverständnis von Abgeordneten während der Krise. Dazu befragen die Studierenden insgesamt 33 Mitglieder des Deutschen Bundestages (19. Wahlperiode) während der ersten Infektionswelle. Ausgehend von der (aus qualitativer Sicht) neuartigen und bisher wenig erforschten Krisensituation gehen die Studierenden offen in die Interviewsituationen, gleichzeitig strukturieren ihre theoretischen Vorkenntnisse der Verwaltungs- und Politikwissenschaft (u. a. bezüglich demokratischer Entscheidungsprozesse, Prozesse parlamentarischer Kommunikation sowie der handlungsbezogenen Repräsentation der Abgeordneten) die Gespräche vor und sind handlungsleitend auch bei der Auswertung der Interviewdaten. Ziel ist eine Analyse der Bedeutung der Krisensituation für den Nexus zwischen Repräsentant:innen und Repräsentierten.

Der Sammelband beinhaltet insgesamt acht Beiträge, die von den Teilnehmer:innen des Forschungsseminars eigenständig verfasst wurden. Sie werden durch eine kurze Einführung und einen Schlussteil des Dozenten gerahmt. Die Texte fokussieren jeweils unterschiedliche Themenaspekte, die sich auf die Wahlkreisarbeit von Bundestagsabgeordneten beziehen (u. a. Organisation der Bundestags‑/Wahlkreisbüros, Repräsentationsverständnis, Kommunikation, Parteiarbeit). Die Beiträge weisen inhaltlich Überschneidungen auf, bauen aber auch aufeinander auf und können nicht gänzlich unabhängig voneinander gelesen werden. Beim Lesen entsteht der Eindruck, dass man sich hinsichtlich der inhaltlichen Strukturierung des Sammelbandes für eine Chronologie und einen Spannungsbogen entschieden hat. Die unterschiedlichen Artikel haben jeweils einen Umfang von knapp 20 Seiten und sind klassisch sozialwissenschaftlich aufgebaut: Nach der Einführung und dem theoretischen Rahmen folgt die Beschreibung und die Interpretation der Interviewdaten. Die Beiträge lesen sich insgesamt kurzweilig, spannungsreich und innovativ (dank des unverstellten Blickes der Autor:innen).

Nach der kurzen Einführung, in der eine einleitende theoretische Verortung hinsichtlich der Problembewältigung während der Krise durch das Parlament sowie das Repräsentations- und Rollenverständnis der Abgeordneten vorgenommen wird, folgt der Beitrag von *Kevin W. Settles*. In diesem Beitrag wird das methodische Vorgehen hinsichtlich der kriterienbasierten Auswahl der Interviewpartner:innen, Durchführung sowie Auswertung der Interviews vorstellt. Das heißt die Grundgesamtheit der Interviewpartner:innen wird hinsichtlich ihrer Sozialstruktur durchleuchtet, der Leitfaden inhaltlich vorgestellt sowie das Analyseraster, mit denen die Interviewdaten strukturiert werden, präsentiert. Das legt die methodologische Grundlage, denn die nachfolgenden Beiträge beziehen sich auf diesen Datensatz. Hinsichtlich der sozialdemografischen Struktur der interviewten Abgeordneten werden Merkmale wie das Geschlecht, die geografische Zuordnung (West/Ost), Mandatstypen (Direkt‑/Listenkandidierenden) sowie die Fraktionszugehörigkeit unterschieden. Die Merkmale dienen einigen Beiträgen als unabhängige Variable, um bestimmte Ausprägungen oder Effekte auf das Repräsentations- und Rollenverständnis zu erklären.

Es folgt zunächst der Beitrag von *Max Follmer*, der sich mit der Fragestellung beschäftigt, wie sich die Organisation der Bundestags- und Wahlkreisbüros unter den Umständen der Krise verändert. Das beinhaltet eine Vielzahl von unterschiedlichen Themenaspekten. Insgesamt geht es darum, dass eine reibungslos funktionierende und resiliente Organisation Voraussetzung dafür ist, dass die Abgeordneten ihrer politischen Arbeit nachgehen können. Interessanterweise gibt es zum jetzigen Zeitpunkt kaum wissenschaftliche Studien zu diesem Thema, was die Bewertung und die Kontextualisierung der Daten erschwert. Schwerpunktmäßig geht es insbesondere um die Umstellung der Büros auf mobile Arbeitsmodelle (mit Unterstützung digitaler Anwendungen) und deren Nachhaltigkeit. Es werden auch die möglichen Unterstützungsleistungen thematisiert, welche die Abgeordneten, u. a. durch die Bundestagsverwaltung sowie durch die jeweilige Fraktion (Bereitstellen von Konferenzdiensten, technischer Support) erhalten haben. Der nachfolgende Beitrag von *Christopher Isensee *beschäftigt sich mit dem Rally-’round-the-Flag-Effekt. Dieser zeigt sich meist zu Beginn einer Krisensituation durch hohe Zustimmungswerten für Politiker:innen der Exekutive. Dabei schwindet zunächst die Bedeutung der Parteien (im Parlament), was ein Regieren in einer parlamentarischen Demokratie herausfordernd gestaltet. Denn es kommt zu Verschiebungen im Gewaltenteilungsprinzip. Das Legitimations- und Kräfteverhältnis zwischen Exekutive und Legislative kann sich verändern, weshalb die Abgeordneten die ihnen zugewiesene Funktionen unter veränderten Rahmenbedingungen wahrnehmen. Der Beitrag untersucht den Effekt mit Blick auf den Deutschen Bundestag und zeigt, wie die jeweiligen Abgeordneten unterschiedlich, aber in einem unausgesprochenen Einverständnis mit den Herausforderungen umgehen. Der Beitrag von *Marcel Gauger* (weiter hinten im Buch) schließt hier inhaltlich an und thematisiert in diesem Zusammenhang noch einmal die These von der Entparlamentarisierung, dabei insbesondere die Handlungsfähigkeit zur parlamentarischen Kontrolle durch die Fraktionen der Opposition, deren Selbstverständnis durch die „Stunde der Exekutiven“ (S. 113) in Krisenzeiten ebenfalls infrage gestellt wird. *Johanna Degering* und *Sahand Shahgholi* beschäftigen sich in ihren Beiträgen mit dem Begriff der Repräsentation, der in der Verwaltungs- und Politikwissenschaft einen hohen Stellenwert in der Bewertung des Rollenverständnisses politischer Mandatsträger:innen sowie des Interaktionsverhältnisses von Bürger:innen und ihren politischen Vertreter:innen hat. *Johanna Degering* legt ihrer Analyse die handlungsbezogene Repräsentation nach Hanna F. Pitkin (1967) zugrunde. Nach diesem normativen Verständnis agieren die Mandatsträger:innen gegenüber den Bürger:innen responsiv, d. h. die Abgeordneten sind für die Interessen und die Bedürfnisse der Bürger:innen offen und berücksichtigen diese in ihrer politischen Arbeit. In Zeiten der Krise wird das Repräsentationsverständnis eines jeden Abgeordneten jedoch einer Prüfung unterzogen. Der Beitrag beschreibt auf Basis der Daten, wie sich das Repräsentationsverständnis in Richtung einer ausgeweiteten Responsivität verschiebt. Gleichzeitig offenbart sich aber auch, dass die Bürger:innen (in den Wahlkreisen) den ausdrücklichen Wunsch nach mehr Orientierung in den turbulenten Zeiten an ihre Vertreter:innen herantragen. Hier schließt der Beitrag von *Sahand Shahgholi* an, die sich mit dem Spannungsfeld von Responsivität und politischer Führung beschäftigt, in denen sich die Abgeordneten bewegen. Die Diskussion zum „clash of culture“ zwischen Politiker:innen und Bürger:innen, der durch die COVID-19-Pandemie erneut deutlich hervorgetreten ist, erhält durch diesen Beitrag eine neue Sichtweise. Es handelt sich jedoch nicht um das Manifestieren einer Negativdiagnose (Stichwort Politiker:innenverdrossenheit), sondern es sind Ansätze für mögliche Veränderungen erkennbar. Der Beitrag von *Bettina Stenkmap* widmet sich schließlich dem Thema der politischen Kommunikation und untersucht, wie sich die Nutzung der Kanäle sozialer Medien, um mit der Wähler:innenschaft zu kommunizieren, während der Pandemie weiter ausweitet. Die Rolle der digitalen Medien ist ebenfalls Thema im Beitrag von *Kevin W. Settles*, insbesondere die Anpassungsstrategien der politischen Parteien, um ihre Arbeit aufrechterhalten und fortsetzen zu können.

Stellenweise hätte man sich mit Blick auf den Sammelband gewünscht, dass die Autor:innen hinsichtlich der Interpretation der Daten mit Blick auf Schlussfolgerungen noch einen Schritt weitergehen (nicht zuletzt mit Blick auf die wissenschaftliche Anschlussfähigkeit). Vermutlich hat die Zurückhaltung mit der eingangs des Sammelbandes geäußerte Methodenkritik hinsichtlich der Limitiertheit des Vorgehens zu tun. Trotzdem: Dem Autor:innenkollektiv gelingt ein Sammelband, der für Verwaltungs- und Politikwissenschaftler:innen gleichermaßen eine Reihe von interessanten zukünftigen Forschungsprämissen bereithält. Teilweise fehlt es einigen Beiträgen an sprachlicher Präzision, Qualitätsunterschiede zwischen den Beiträgen sind marginal erkennbar. Das Werk zeigt, dass die Nähe zu aktuellen politischen Phänomenen und ihre wissenschaftliche Bewertung eine besondere Qualität hat. Man wünscht den Teilnehmer:innen des Forschungsseminars, dass sie ihre Themen im Rahmen einer anschließenden Forschungsarbeit und in der Praxis weiter vertiefen.

